# Two Cases of Rectal Xanthoma Presenting as Yellowish to Whitish Lesions during Colonoscopy

**DOI:** 10.1155/2017/5975107

**Published:** 2017-06-01

**Authors:** Masaya Iwamuro, Takehiro Tanaka, Daisuke Takei, Yuusaku Sugihara, Keita Harada, Sakiko Hiraoka, Yoshiro Kawahara, Hiroyuki Okada

**Affiliations:** ^1^Department of Gastroenterology and Hepatology, Okayama University Graduate School of Medicine, Dentistry and Pharmaceutical Sciences, Okayama 700-8558, Japan; ^2^Department of General Medicine, Okayama University Graduate School of Medicine, Dentistry and Pharmaceutical Sciences, Okayama 700-8558, Japan; ^3^Department of Pathology, Okayama University Hospital, Okayama 700-8558, Japan; ^4^Department of Endoscopy, Okayama University Hospital, Okayama 700-8558, Japan

## Abstract

Two cases of rectal xanthomas are described. One case is that of a 56-year-old Japanese man in whom multiple yellowish spots measuring approximately 3 to 5 mm were observed in the rectum during colonoscopy. The other case is that of a 78-year-old Japanese man in whom colonoscopy showed a whitish plaque of 4 mm in diameter in the rectum. Biopsy examinations performed on both patients revealed the deposition of xanthoma cells within the rectal mucosa. Within the gastrointestinal tract, xanthomas most frequently arise in the stomach, whereas the colorectum is rarely affected. Despite this infrequency, the two cases indicate that xanthomas should be recalled when yellowish to whitish lesions are observed in the colorectum.

## 1. Introduction

Xanthomas in the alimentary tract are benign mucosal lesions resulting from the aggregation of foamy histiocytes within the gastrointestinal mucosa [1]. Within the gastrointestinal tract, the stomach is the most frequently affected by xanthoma, whereas other parts such as the esophagus, duodenum [2], small intestine, and colorectum usually remain unaffected. Macroscopic features of gastric xanthoma are well known as yellow to white well-demarcated plaques or nodules [3]. Endoscopic images of colorectal xanthoma have rarely been reported in the literature due to their infrequency.

We recently encountered two patients with rectal xanthomas that were observed as yellowish to whitish lesions during colonoscopy. In this report, we focus mainly on the macroscopic characteristics of the colorectal xanthoma and review previously reported cases of this disease.

## 2. Case Report

### 2.1. Case  1

A 56-year-old Japanese man was referred to our hospital for investigation of tarry stool. The patient had been consuming lansoprazole, irsogladine, metoprolol, flutoprazepam, and ethyl loflazepate for gastritis, hypertension, and anxiety disorder but had no history of dyslipidemia or diabetes mellitus. A physical examination revealed no abnormalities in his abdomen or xanthomas on his skin, and laboratory findings showed no abnormalities. The levels of cholesterol, triglyceride, and plasma glucose were within normal range. Esophagogastroduodenoscopy showed erosive and atrophic gastritis.

**Figure 1:**
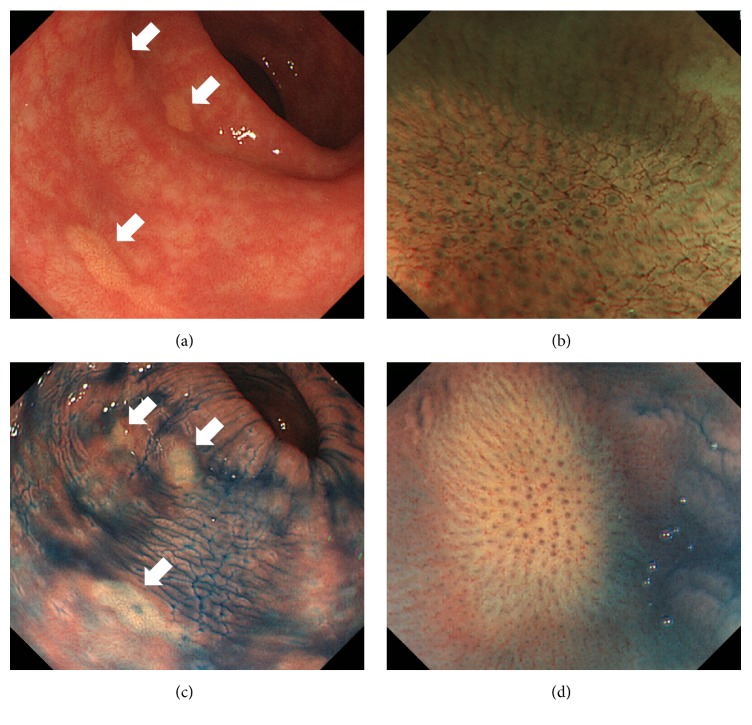
Colonoscopy images of case  1. Multiple yellowish spots measuring approximately 3 to 5 mm are seen in the rectum (a). Magnifying observation with narrow-band imaging reveals intact pits of the rectal mucosa (b). Indigo-carmine spraying emphasizes the whitish to yellowish color of the lesions (c, d).

During colonoscopy, multiple yellowish spots measuring approximately 3 to 5 mm were observed in the rectum, in addition to hemorrhoids (Figure 1(a)). Magnifying observation with narrow-band imaging revealed that the pits of the rectal mucosa were intact (Figure 1(b)). Indigo-carmine spraying emphasized the whitish to yellowish color of the lesions (Figures 1(c) and 1(d)). Histological analysis of the biopsied samples revealed accumulation of xanthoma cells within the mucosal layer. Consequently, a diagnosis of rectal xanthoma was made.

### 2.2. Case  2

A 78-year-old Japanese man had been treated for remitting seronegative symmetrical synovitis with pitting edema. The patient underwent colonoscopy for screening purposes. He had been taking 2 mg/day of prednisone but had no history of dyslipidemia or diabetes mellitus. A physical examination revealed no xanthomas on his eyelid or extremities and a blood test revealed that his levels of cholesterol, triglyceride, and plasma glucose were within normal range.

**Figure 2:**
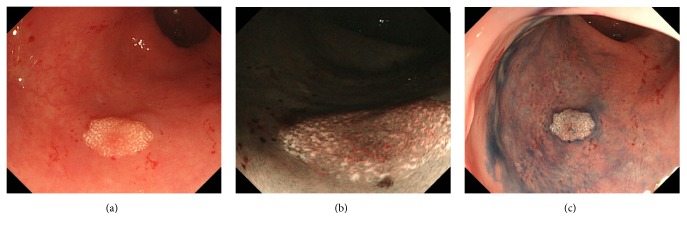
Colonoscopy images of case  2. A whitish plaque of 4 mm in diameter in the rectum is seen (a). Narrow-band imaging (b) and indigo-carmine spraying (c) show whitish lesions more clearly.

**Figure 3:**
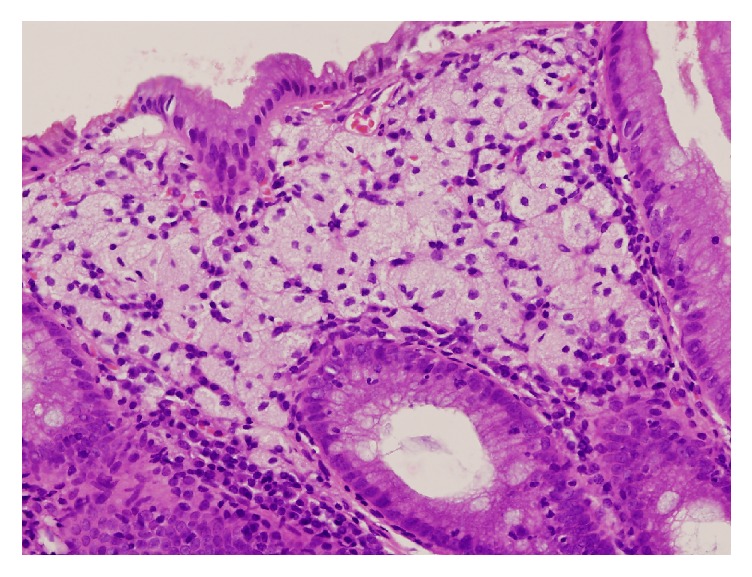
Pathological image of case  2. Biopsy examination reveals massive deposition of xanthoma cells within the rectal mucosa.

Colonoscopy showed a whitish plaque of 4 mm in diameter in the rectum (Figure 2). Biopsy examination revealed massive deposition of xanthoma cells within the rectal mucosa (Figure 3), leading to the diagnosis of rectal xanthoma.

## 3. Discussion

In the two cases presented, rectal xanthomas were observed as yellowish to whitish lesions during colonoscopy. As described above, typical gastric xanthomas are well-demarcated whitish plaques or nodules. Similarly, cases with xanthomas in the sigmoid colon and rectum, showing multiple, well-defined, and whitish-yellow lesions, have been previously reported [4, 5]. Weinstock et al. reported a case consistent with xanthoma presenting as flat, yellow, and irregularly shaped lesions in the sigmoid colon [6]. Endoscopic images presented in their report show a hexagonal-shaped appearance, which is not similar to the macroscopy of gastric xanthomas. The case report by Moran and Fogt, which did not present endoscopic images, described a xanthoma in the rectosigmoid colon as polypoid in appearance [7]. Miliauskas et al. reported that, by reviewing their four cases and nine previously reported cases, papules were observed in eight cases and polyps were noted in three cases [8, 9]. Nakasono et al. summarized 28 colorectal xanthomas biopsied from 25 patients. Xanthomas were located in the sigmoid colon (17/28 lesions) and rectum (11/28 lesions) [9]. Macroscopically, 23 lesions presented sessile appearance and the remaining five lesions were pedunculated. Twelve of the xanthoma lesions were reddish, five were whitish, two were yellow-whitish, and one was normal color. Consequently, the morphology of colorectal xanthomas varies from flat, sessile to pedunculated lesions, with yellowish, yellow-whitish, whitish, and even reddish colors. Endoscopists must recall this entity as a differential diagnosis when they observe whitish lesions in the colorectum regardless. In the two cases we presented, narrow-band imaging highlighted lesions that were yellowish to whitish in color. Moreover, observation under magnification revealed intact pits in the rectal mucosa. We propose that the intact pits reflect undamaged epithelial cells observed by histopathological examination of biopsy specimens. However, since this observation is based on only two cases, usefulness of observation under magnification and optical chromoendoscopy techniques such as narrow-band imaging, flexible spectral imaging color enhancement, and i-SCAN for the diagnosis of colorectal xanthomas should be further investigated.

Diseases other than xanthomas that present with yellowish to whitish lesions in the colon include pseudomembranous colitis, lipomas, and lymphomas. Pseudomembranous colitis is a common cause of antibiotic-associated diarrhea. This disease is characterized by elevated yellow-white plaques that coalesce to form pseudomembranes along the colorectal mucosa and can be easily diagnosed based on their endoscopic appearance [10]. Colonic lipomas are generally observed as solitary, soft, spherical, smooth yellowish lesions [11]. Pedunculated and semipedunculated lipomas can be easily diagnosed, but those with a slightly elevated appearance may be misinterpreted as xanthomas. Lymphomas, particularly the indolent subtypes, sometimes present as whitish, slightly elevated lesions in the colorectum. For instance, follicular lymphomas in the colorectum are identified as papular, polypoid, or flat elevated lesions [12]. While the color of the lymphoma lesions is not grossly different than that of the surrounding intact mucosa, colorectal xanthomas, as observed in this report, do show a definite color contrast between the lesions and surrounding mucosa.

Microscopically, foamy macrophages are generally confined to the lamina propria mucosae of the colorectum; the muscularis mucosae or submucosa is rarely affected. Nakasono et al. reported that hyperplastic change was identified in the surface epithelium in 22/28 lesions [9]. In addition, thickening of the basement membrane of the surface epithelium, cell debris, and proliferation of the capillaries were frequently observed. In contrast, the two presented cases lack hyperplastic change in the epithelium. In case 2, we speculate that the accumulation of foamy cells itself accounts for the slightly elevated morphology since prominent deposition of foamy cells exists in the lamina propria mucosae (Figure 3).

Although cutaneous and tendinous xanthomas occur in relation to hyperlipidemia, gastrointestinal xanthomas are not associated with dyslipidemia [3, 7, 13]. Miliauskas et al. reported that, among four cases with colorectal xanthomas, none had hyperlipidemia and only one had diabetes mellitus [7, 8]. The two cases presented did not have metabolic disorders, including increased lipid levels and diabetes mellitus. Gastrointestinal xanthomas are asymptomatic and believed to be harmless [8, 9]. Therefore, no specific treatment is considered necessary for colorectal xanthomas.

The etiology of colorectal xanthomas remains unknown. Xanthomas in the stomach are assumed to arise as an inflammatory response to focal mucosal damage and chronic injury such as chronic gastritis [3, 7, 14–16]. The resident macrophages commonly exist in the subepithelial lamina propria of the gastrointestinal tract. Although macrophages are not pathologically noticeable in normal gut mucosa, they can be identified when they phagocytize and accumulate exogenous or endogenous substances [17]. Content of the foamy macrophages in xanthomas is assumed to come from lipids derived from damaged cell membranes [3, 7, 14]. Mucosal damage and chronic injury are believed to be associated with the pathogenesis of colorectal xanthomas [7, 15]. Toxic factors, focal infection, or mechanical damage by peristalsis or contact with feces may cause such injury to the colorectal mucosa [5, 9].

In conclusion, we encountered two patients with rectal xanthomas. Both cases showed yellow to whitish lesions in the rectum. Although colorectal xanthoma can present varied morphology, endoscopists should consider this entity when yellowish to whitish lesions are observed in the colorectum.
